# Supplementary material for: Comparative transcriptome provides molecular insight into defense-associated mechanisms against spider mite in resistant and susceptible common bean cultivars
Source: PLoS One. 2020 Feb 4;15(2):e0228680. doi: 10.1371/journal.pone.0228680 (PMC6999899; doi:10.1371/journal.pone.0228680)
Supplement: Attachment — Submitted filename: PONE-D-19-29263_reviewer (1).pdf [file pone.0228680.s009.pdf]

PLOS ONE

Comparative transcriptome provides molecular insight into defense-associated mechanisms against spider mite in resistant and susceptible common bean cultivars

--Manuscript Draft--

**Manuscript Number:**	PONE-D-19-29263
**Article Type:**	Research Article
**Full Title:**	Comparative transcriptome provides molecular insight into defense-associated mechanisms against spider mite in resistant and susceptible common bean cultivars
**Short Title:**	Transcriptome analysis of common bean attacked by Spider mite
**Corresponding Author:**	Aboozar Soorni<br>Isfahan University of Technology<br>Isfahan, IRAN, ISLAMIC REPUBLIC OF
**Keywords:**	
**Abstract:**	Common bean (Phaseolus vulgaris L.) is a major source of proteins and one of the most important edible foods for more than three hundred million people in the world. The common bean plants are frequently attacked by Spider mite (Tetranychus urticae Koch), leading to a significant decrease in plant growth and economic performance. The use of resistant cultivars and the identification of the genes involved in plant-mite resistance are practical solutions to this problem. Hence, a comprehensive study of the molecular interactions between resistant and susceptible common bean cultivars and spider mite can shed light into the understanding of mechanisms and biological pathways of resistance. In this study, one resistant (Naz) and one susceptible (Akhtar) cultivars were selected for a transcriptome comparison at different time points (0, 1 and 5 days) after spider mite feeding. The comparison of cultivars in different time points revealed several key genes, which showed a change increase in transcript abundance via spider mite infestation. These included genes involved in flavonoid biosynthesis process; a conserved MYB-bHLH-WD40 (MBW) regulatory complex; transcription factors (TFs) TT2, TT8, TCP, Cys2/His2-type and C2H2-type zinc finger proteins; the ethylene response factors (ERFs) ERF1 and ERF9; genes related to metabolism of auxin and jasmonic acid (JA); pathogenesis-related (PR) proteins and heat shock proteins.
**Order of Authors:**	Abdul Hadi Hoseinzadeh
	Aboozar Soorni
	Marie Shoorooei
	Masoud Torkzadeh Mahani
	Reza Maali Amiri
	Hossein Allahyari
	Rahmat Mohammadi
**Additional Information:**	
**Question**	**Response**
**Financial Disclosure**<br><br>Enter a financial disclosure statement that describes the sources of funding for the work included in this submission. Review the submission guidelines for detailed requirements. View published research articles from *PLOS ONE* for specific examples.	The authors received no specific funding for this work.

This statement is required for submission and **will appear in the published article** if the submission is accepted. Please make sure it is accurate.<br><br>**Unfunded studies**<br>Enter: *The author(s) received no specific funding for this work.*<br><br>**Funded studies**<br>Enter a statement with the following details:<br>• Initials of the authors who received each award<br>• Grant numbers awarded to each author<br>• The full name of each funder<br>• URL of each funder website<br>• Did the sponsors or funders play any role in the study design, data collection and analysis, decision to publish, or preparation of the manuscript?<br>• **NO** - Include this sentence at the end of your statement: *The funders had no role in study design, data collection and analysis, decision to publish, or preparation of the manuscript.*<br>• **YES** - Specify the role(s) played.<br><br>* typeset	
**Competing Interests**<br><br>Use the instructions below to enter a competing interest statement for this submission. On behalf of all authors, disclose any competing interests that could be perceived to bias this work—acknowledging all financial support and any other relevant financial or non-financial competing interests.<br><br>This statement **will appear in the published article** if the submission is accepted. Please make sure it is accurate. View published research articles from *PLOS ONE* for specific examples.	The authors have declared that no competing interests exist.

Powered by Editorial Manager® and ProduXion Manager® from Aries Systems Corporation

**NO authors have competing interests**<br><br>Enter: *The authors have declared that no competing interests exist.*<br><br>**Authors with competing interests**<br><br>Enter competing interest details beginning with this statement:<br><br>*I have read the journal's policy and the authors of this manuscript have the following competing interests: [insert competing interests here]*<br><br>* typeset	
**Ethics Statement**<br><br>Enter an ethics statement for this submission. This statement is required if the study involved:<br><br>• Human participants<br>• Human specimens or tissue<br>• Vertebrate animals or cephalopods<br>• Vertebrate embryos or tissues<br>• Field research<br><br>Write "N/A" if the submission does not require an ethics statement.<br><br>General guidance is provided below. Consult the submission guidelines for detailed instructions. **Make sure that all information entered here is included in the Methods section of the manuscript.**	N/A

Powered by Editorial Manager® and ProduXion Manager® from Aries Systems Corporation

**Format for specific study types**<br><br>**Human Subject Research (involving human participants and/or tissue)**<br>• Give the name of the institutional review board or ethics committee that approved the study<br>• Include the approval number and/or a statement indicating approval of this research<br>• Indicate the form of consent obtained (written/oral) or the reason that consent was not obtained (e.g. the data were analyzed anonymously)<br><br>**Animal Research (involving vertebrate animals, embryos or tissues)**<br>• Provide the name of the Institutional Animal Care and Use Committee (IACUC) or other relevant ethics board that reviewed the study protocol, and indicate whether they approved this research or granted a formal waiver of ethical approval<br>• Include an approval number if one was obtained<br>• If the study involved *non-human primates*, add *additional details* about animal welfare and steps taken to ameliorate suffering<br>• If anesthesia, euthanasia, or any kind of animal sacrifice is part of the study, include briefly which substances and/or methods were applied<br><br>**Field Research**<br><br>Include the following details if this study involves the collection of plant, animal, or other materials from a natural setting:<br>• Field permit number<br>• Name of the institution or relevant body that granted permission	
**Data Availability**<br><br>Authors are required to make all data underlying the findings described fully available, without restriction, and from the time of publication. PLOS allows rare exceptions to address legal and ethical concerns. See the PLOS Data Policy and FAQ for detailed information.	Yes - all data are fully available without restriction

Powered by Editorial Manager® and ProduXion Manager® from Aries Systems Corporation

A Data Availability Statement describing where the data can be found is required at submission. Your answers to this question constitute the Data Availability Statement and **will be published in the article**, if accepted.<br><br>**Important:** Stating 'data available on request from the author' is not sufficient. If your data are only available upon request, select 'No' for the first question and explain your exceptional situation in the text box.<br><br>Do the authors confirm that all data underlying the findings described in their manuscript are fully available without restriction?	
**Describe where the data may be found in full sentences. If you are copying our sample text, replace any instances of XXX with the appropriate details.**<br><br>• If the data are **held or will be held in a public repository**, include URLs, accession numbers or DOIs. If this information will only be available after acceptance, indicate this by ticking the box below. For example: *All XXX files are available from the XXX database (accession number(s) XXX, XXX.*).<br>• If the data are all contained **within the manuscript and/or Supporting Information files**, enter the following: *All relevant data are within the manuscript and its Supporting Information files.*<br>• If neither of these applies but you are able to provide **details of access elsewhere**, with or without limitations, please do so. For example:<br><br>*Data cannot be shared publicly because of [XXX]. Data are available from the XXX Institutional Data Access / Ethics Committee (contact via XXX) for researchers who meet the criteria for access to confidential data.*<br><br>*The data underlying the results presented in the study are available from (include the name of the third party*	All RNA-Seq data were deposited to the NCBI sequence read archive (SRA) under the project PRJNA482175.

and contact information or URL).<br>• This text is appropriate if the data are owned by a third party and authors do not have permission to share the data.<br><br>* typeset	
Additional data availability information:	

Click here to download Manuscript Manuscript_PLOS_ONE.docx

Comparative transcriptome provides molecular insight into defense-associated mechanisms against spider mite in resistant and susceptible common bean cultivars

Abdul Hadi Hoseinzadeh[1*¶] , Aboozar Soorni[2*¶], Marie Shoorooei[1], Masoud Torkzadeh Mahani[3], Reza Maali Amiri[1], Hossein Allahyari[4] and Rahmat Mohammadi[1]

[1]Department of Agronomy and Plant Breeding, Faculty of Agriculture, University of Tehran, Karaj, Iran

[2]Department of Biotechnology, College of Agriculture, Isfahan University of Technology, Isfahan 84156-8311, Iran

[3]Department of Biotechnology, Institute of Science, High Technology and Environmental Science, Graduate University of Advanced Technology, Kerman, Iran

[4]Department of Plant Protection, Faculty of Agriculture, University of Tehran, Karaj, Iran

***Corresponding author.**

Abdul Hadi Hoseinzadeh: ahzadeh@ut.ac.ir
Aboozar Soorni: soorni@cc.iut.ac.ir

[¶]These authors contributed equally to this work

Abstract

Common bean (*Phaseolus vulgaris* L.) is a major source of proteins and one of the most important edible foods for more than three hundred million people in the world. The common bean plants are frequently attacked by Spider mite (*Tetranychus urticae* Koch), leading to a significant decrease in plant growth and economic performance. The use of resistant cultivars and the identification of the genes involved in plant-mite resistance are practical solutions to this problem. Hence, a comprehensive study of the molecular interactions between resistant and susceptible common bean cultivars and spider mite can shed light into the understanding of mechanisms and biological pathways of resistance. In this study, one resistant (*Naz*) and one susceptible (*Akhtar*) cultivars were selected for a transcriptome comparison at different time points (0, 1 and 5 days) after spider mite feeding. The comparison of cultivars in different time points revealed several key genes, which showed a change increase in transcript abundance via spider mite infestation. These included genes involved in flavonoid biosynthesis process; a conserved *MYB-bHLH-WD40* (MBW) regulatory complex; transcription factors (TFs) TT2, TT8, TCP, Cys2/His2-type and C2H2-type zinc finger proteins; the ethylene response factors (ERFs) ERF1 and ERF9; genes related to metabolism of auxin and jasmonic acid (JA); pathogenesis-related (PR) proteins and heat shock proteins.

Keywords: Common bean, Spider mite, Transcriptome, Defense mechanisms, Biological pathways.

Introduction

Common bean (*Phaseolus vulgaris* L.), is one of the most important edible foods in the world which provides about 50% of the grain legumes for direct human consumption [1-3]. In addition, it is an inexpensive healthy food due to having the richest sources of proteins (20-25%), micronutrients and calories [2]. Common bean is widely distributed around the world. In Asia, most collections exist in India [4], and Iran [5]. Notably, over the last 10 years, the production of common bean has increased ~33% in Asia [2]. Because of high nutrient content and commercial potential, common bean holds great promise for fighting hunger and increasing income. Low yield of this crop is attributed to pest attack, weak soil fertility, drought and salinity, and poor agronomic practices [6]. According to the records, *Tetranychus urticae* Koch (TSSM) is the most widespread and the most polyphagous herbivores mites which feed on cell contents of common bean and causes serious substantial economic losses (up to 100% yield losses) in fields and greenhouses [7, 8]. The TSSM damages plant cells by its stylet that pierces the leaf either in between epidermal pavement cells or through a stomatal opening, suck-out their contents and forms the chlorotic lesions at the feeding sites [8, 9]. In recent years, it has become evident that insect-resistant crops have brought great benefits, not only in terms of economic, but also because of the reduction of pesticides use and keeping a safe environment. The development of new cultivars is being established as one of the most appropriate methods and the main objective of plant breeding programs for resistance to TSSM [10, 11]. However, lacking information on how plant and mite interact with each other emphasizes the importance of a comprehensive study of the molecular interactions between common bean and *T. urticae* to understand the mechanisms and potential biological pathways of common bean resistance. Although RNA-Seq has been used to study the expression profiles of stress response genes in model and non-model plants, but there has not been any study of common bean transcriptome changes due to spider mite feeding. In this study we used RNA-seq [illegible]lysis to detect differences in gene expression between two cultivars of *P. vulgaris* (susceptible and resistant), and specify effective genes and pathways in response to *T. urticae* infestation. Such information could lead to identity resistant mechanisms and genes in common bean and improve the breeding efforts by identifying molecular markers to incorporate resistance into commercial bean varieties.

Materials and Methods

Plants and insect infestation

According to our previous study [12], two cultivars, including *Akhtar* and *Naz* were selected as susceptible and resistant cultivars to *T. urticae*, respectively. The experiment was conducted using a factorial experiment based on completely randomized design with three replicates in greenhouse condition (28 ± 3°C temperature, 40-50% relative humidity, photoperiod 16h light and 8h darkness). In six-leaf stage of Meier [13], 45 same-aged adult female mites were placed on sixth leaf of cultivars. The leaves were collected after 0, 1 and 5 days of infestation. Treated leaves were frozen in liquid nitrogen and kept at −80 °C until they were used for RNA extraction.

RNA isolation and transcriptome sequencing

Total RNA was extracted from two biological replicates, which each were pooled samples from at least three plants using TRIzol® Reagent (Invitrogen) as described by the manufacturer's protocol, and then treated with RNase-free DNaseI (Invitrogen). Nanodrop™ 2000 spectrophotometer, agarose gel electrophoresis and Bioanalyzer 2100 (Agilent) were used to check and confirm quantity and quality of RNAs. All RNAs were sent to Beijing Genomic Institute (BGI) in China for library preparation and transcriptome sequencing using the Illumina HiSeq 2500 platform to generate Paired-end (2×150 bp) reads.

Reads preprocessing and differentially expression analysis

~~The raw reads were downloaded from BGI institute web site in Fastq format~~ and then deposited in the NCBI SRA database under the project PRJNA482175. All RNA-Seq data were subjected to quality control (QC) analysis using Trimmomatic software [14] to ~~drop out~~ low quality reads, adapters and other Illumina-specific sequences, minimum length 50 bp and minimum quality 30 determined as quality thresholds. Before and after filtering, the quality of the raw sequences was assessed with FastQC (https://www.bioinformatics.babraham.ac.uk/projects/fastqc/). Clean reads were mapped to the *P. vulgaris* [15] (https://phytozome.jgi.doe.gov[illegible]d *T.urticae* [16] reference genomes V. 2.0 via RNA-seq aligner STAR software [17] requiring at least 90% of the read sequence to match with at least 95% identity. The STAR-resultant *.bam* files were used to

estimate the abundance of mapped reads, differential expression analyses, and visualization of analyses results using Cufflinks [18] package coupled with CummeRbund [19]. Cufflinks was used to calculate FPKM values and differential expression analysis was done with Cuffdiff. The analysis focused on genes with statistically significant difference in expression levels between times and cultivars. The genes were considered significantly differentially expressed if false discovery rate (FDR, the adjusted P value) was <0.01 and Log2 FPKM (fold change) was ≥1.0.

GO Enrichment Analysis

The functions of the DEGs were characterized using AgriGO's Singular Enrichment Analysis (SEA) module to identify the enriched Gene Ontology terms (http://bioinfo.cau.edu.cn/agriGO/analysis.php) with the agriGO database [20]. The enrichment analysis was performed at significance level of 0.05.

Quantitative real-time PCR validation

To validate candidate differentially expressed genes (DEGs), qRT-PCR was performed for six DEGs and *Actin 11* as a reference gene with three replicates. Primers were designed by Primer 3.0 [21] (Table 1), and cDNAs were synthesized by using TaKaRa cDNA Synthesis Kit (TaKaRa, Dalian, China) according to the manufacturer's instructions. The 20 μL qRT-PCR solutions contained EvaGreen Master Mix (Solis Biodyne, 5x), 0.3 μL forward and 0.3 μL reverse primers, and 30 ng of cDNA template. qRT-PCR reactions (95 °C, 3 min; 95 °C, 5 s; 60 °C, 34 s; 40 cycles) were carried out on a Bio-Rad iQ5 Optical System (Bio-Rad Laboratories, CA, USA). Finally, relative gene expression was calculated using $2^{-\Delta\Delta Ct}$ formula and REST software [22].

Table 1. Description of the candidate ~~gens~~ and primer sequences for qRT-PCR assayefficiencies.

Funcional annotation	Gene ID	Forward primer	Reverse prime
Pathogenesis-related protein	Phvul.004G155500	TGGGATACAGCTACAGCATCGT	ATCTTCATTGGGTGGAGCATCT
WRKY transcription factor 50	Phvul.009G080000	GTCGCTGAGATCGGAGAATC	GCAAATCCAGCTTTGACCAT
Heat shock protein (Molecular chaperone)	Phvul.008G011400	CTTTCAACACCAACGCCATG	GCTCAAGCTCCGAGTAGG

Leucine Rich Repeat	Phvul.008G044600	CTTGACTATGAGCTTGTCCCC	TGCTTTCTCTGTAAGGTGTCC
MYB113	Phvul.008G038200	GTCGCTGAGATCGGAGAATC	GCAAATCCAGCTTTGACCAT
Xyloglucan endotransglucosylase/hydrolases	Phvul.005G111300	AGTTCGACGAGCTGTTCCAG	ACGTTGGTCTGCACGCTGTA

Results and Discussion

Quality control and mapping statistics

A total of 12 RNA libraries were sequenced with the ~~sequencing depth~~ ranging from 26.8–30.2 million paired-end reads (Table 2). Approximately 70-73% of reads passed the quality control and an 84.58-90.30% of the clean reads were mapped to unique location in the common bean reference genome. Alignment of clean reads to *T. urticae* reference genome was also carried out to determine whether a significant mite RNA contamination exists in our datasets. Assessment of quality of mRNA-seq data revealed less than 0.1% mapping, indicating a strong enrichment of genes specific for *P. vulgaris* in all samples.

Table 2. Summary of sample information and transcriptome sequengcing output statistics for the RNA-seq libraries

Cultivars	Replicate	Time points	Reads before quality control	Reads after quality control	Removing percent
Akhtar (susceptible)	Replicate 1	Control	30261354	21020563	30.54
		1 Day	28834008	20871380	27.62
		5 Day	26832585	19131482	28.70
	Replicate 2	Control	31261254	22020354	29.56
		1 Day	27836208	20971682	24.66
		5 Day	26632382	19231180	27.79
Naz (resistan[illegible]	Replicate 1	Control	30593227	22305624	27.09
		1 Day	27677349	19152514	30.80
		5 Day	29214322	21461892	26.54
	Replicate 2	Control	30243118	22212456	26.55
		1 Day	28123454	19252314	31.54
		5 Day	28876322	21863542	24.29

Distribution of differentially expressed genes (DEGs)

Differentially expressed genes analysis was performed for the pairwise comparisons of twelve libraries. The largest differences in expression occurred among three time points of resistant cultivar. When comparing the different time points for resistant cultivar, 274 differentially expressed genes were identified (Table S1), almost the same number of DEGs for susceptible cultivar (270 DEGs, Table S2). The number of up-regulated genes was higher than down-regulated genes in all different time point comparisons in both cultivars. To gain a better understanding, the overlap differentially expressed patterns of DEGs were analyzed between cultivars in each time point and across time points in each cultivars using Venn diagram. The comparison of cultivars in each time points [illegible]vealed 48, 65 and 81 up-regulated genes along with 46, 59, 68 down-regulated genes in resistant cultivar for control samples and these infested at 1 and 5 days post-feeding, respectively (Fig 1, Table S3-5). The number of DEGs showed a rising trend with the extension of infestation time, So that the smallest and largest differences were observed between resistant and susceptible plants at first and third time points, in which 94 and 146 DEGs were identified, respectively. This result indicates there are probably no significant differences in gene expression patterns during the first attempts of spider mite in both susceptible and resistant reactions. However, gene expression patterns were more different during the second phase of infestation depending on the resistance/susceptibility of the plant.

Fig 1. Venn diagram showing the number of specific, ~~commonalities and differences~~ DEGs between pair time points in both cultivars. Up_T0, genes upregulated in resistant cultivar in comparison with susceptible cultivar at first time point. Down_T0, genes downregulated in resistant cultivar or upregulated in susceptible cultivar at first time point. T1 and T5 represent the second and third time points, respectively.

Gene expression patterns were also different with the extension of infestation time depending on the resistance/susceptibility of common bean cultivars. Among DEGs, approximately 44% and 37% of up-regulated genes were common among three time points, while less than 7% of down-regulated genes were shared among times in both cultivars. Interestingly, there was no any common up or down regulated gene between T0T1 (comparison of first and second time points) and T1T5 (comparison of second and third time points) and also any unique gene for T0T5 (comparison of first and third time points). As shown in the Venn diagram in Fig 2, the number of uregulated DEGs in T0T5 comparision was higher than T0T1 comparision in both cultivars.

This analysis indicated that more than 70% and 85% of DEGs were common between T0T1 and T0T5 comarisions, respectively.

Fig 2. Ve[illegible] diagram representing ~~commonly and specifically~~ up- and down-regulated genes between two sets in each cultivar. Pink circle (first set) represents the comparision of first and second time points and green (second set) is the comparision of first and third time points. Up and Down represent upregulated, downregulated genes. Res and Sus represent resistant and susceptible cultivars.

Functional Classification and GO enrichment analysis of DEGs

To eliminate the effects of genetic differences, DEGs were compared between cultivars at the same time points. GO analysis detected 57, 39 and 46 categories, according to biological process (P), molecular function (F), and cellular component (C) among all up- and down-regulated genes at time 0, 1 and 5, respectively.

Secondary Metabolism

The differences between control samples was determined by the metabolism of phenylpropanoid, a central [illegible] secondary metabolite production of defense-related compounds [illegible]3-25], including anthocyanins and flavonoids (Fig 3). The two most induced genes involved in flavonoid biosynthesis process corresponded to dihydroflavonol reductase (DFR) and chalcone synthase (CHS). We found transcripts of DFR which were up-regulated in resistant cultivar (Fig 4). DFR has been previously reported as induced by *Fusarium oxysporum* inoculations on *Linum usitatissimum* [26]. *Colletotrichum camelliae* on *Camellia sinensis* [27] and *Elsinoe ampelina* in *grapevine* [illegible]]. DFR is also a key regulatory poin[illegible]elonging to the subgroup of late anthocyanin biosynthesis genes which can be activated by TFs such as MYBs [29, 30]. CHS showed more than 7-fold change increase in resistant cultivar. O-methyltransferases (OMTs), involved in phenylpropanoids, flavonoids, and anthocyanin methylation, was up-regulated in resistant cultivar and showed an 8-fold increase in expression level upon spider mite feeding. O-Methylation plays key roles in plant defense following pathogen attack [31]. Additionally, the interaction network suggests the possible involvement of *CYP* genes (CYP72A7 and CYP71A26) in resistance, previously reported in several studies [32, 33]. In the susceptible genotype, CYP83B1 gene required for the synthesis of indole glucosinolates, was down-

regulated during infestation which is not consistent with previous reports in a number of researches [26, 34]. Finally, other secondary metabolism gene (BAS: beta-amyrin synthase) with higher transcript abundance in resistant cultivar was related to sesquiterpenoid and triterpenoid biosynthesis.

Fig 3. GO enrichment analysis for up-regulated genes in resistant cultivar as compared to susceptible cultivar at day 0 (control samples). Boxes in the graph represent GO IDs, term definitions and statistical information. Significant GO terms ($p \leq 0.05$) are marked with color. The degree of color saturation of a box is positively correlated to the enrichment level of the term.

Fig 4. Interaction networks of up-regulated genes identified in resistance cultivar as compared to susceptible cultivar at day 0 (control samples).

Transcriptional Regulation

TF families found in our study are widely reported to be involved in plant defense responses, including MYB, WRKY, ethylene responsive factors (ERFs), zinc finger domain proteins and basic helix-loop-helix (bHLH). A key TF that currently appears in the studies of plant-pathogen interactions [26, 35] and had a significant expression in resistant cultivar of our study is MYB113. *TRANSPARENT TESTA4* ([illegible]T4), a chalcone and stilbene synthase family protein, is a key enzyme involved in the biosynthesis of flavonoids to encode chalcone synthase (CHS), and is required for the accumulation of purple anthocyanins in leaves and stems [36]. TT4 along with TRANSPARENT TESTA8 (TT8), a bHLH DNA-binding superfamily protein which is required for normal expression of DFR [37] associated with MYB113 in the interaction network. An important candidate TF for spider mite resistance is WD40 protein, which was expressed only during mite infestation in the resistant cultivar. Many studies have shown that a conserved *MYB-bHLH-WD40* ([illegible]BW) regulatory complex control the expression of anthocyanin biosynthesis genes [38]. So, we deduced that MBW regulatory complex has the same function in common bean and is involved in resistance. In addition, another TF for spider mite resistance may be TCP protein, ~~whose~~ expression was increased during spider mite infestation in the resistant cultivar. Recent studies suggested that TCP proteins play an important role in systemic acquired resistance (SAR) which is induced plant immunity, activated by pathogen infection [39-41].

Additionally, we identified a gene encoding WRKY50 which was significantly up-regulated in resistant cultivar during the middle-to-late stages (at day 5) of spider mite feeding. The spider mite infestation also affected the expression levels of Cys2/His2-type and C2H2-type zinc finger proteins as up-regulated genes in resistance and susceptible cultivars at day 5 post-infestation, respectively. The Cys2/His2-type zinc finger proteins are not only related to plant stress responses, but also enhance the resistance against pathogen infection [42]. ERFs, another important group of TFs, which play roles in integrating ET/JA signals [30, 43], activating the phenylpropanoid biosynthetic pathway and expression of resistance genes [44, 45], were activated in resistance cultivar at day 5 post-infestation. We observed increased transcript abundance of two key ERFs, including ERF1 and ERF9. The role of ERF1 as a regulator of ethylene responses after pathogen attack has been documented in *Arabidopsis* [46] but ERF9 has not proven to be relevant in the defense responses.

Hormone Regulation

GO enrichment analysis showed other GO terms that significantly overrepresented among up-regulated genes of resistant cultivar, including “response to stress”, “response to stimulus” and “response to jasmonic acid”. JA signaling has closely been associated with defense mechanisms against pathogens and insects [47, 48]. In our study, 13S-lipoxygenase 2 (LOX2), [illegible]A signaling and biogenesis gene, was detected as up-regulated gene in resistant cultivar, where it had a low transcript abundance in susceptible cultivar. Up-regulation of allene oxide synthase (AOS), needed to JA production, at day 1 post-feeding suggests that common bean resistance to the disease is enhanced by the activation of JA signaling pathways. Our result is corroborated with the previous studies where the expression levels of *LOX* and *AOS* significantly increased after infestation [26, 49]. In addition, the expression of two auxin signaling pathway genes, *SAUR* (small auxin-up RNA) and *ARF5* (Auxin response factor 5), were down- and up-regulated in resistant cultivar, respectively. *SAUR* genes are related to cell division[50, 51] and reportedly regulated by the auxin level, indicating that this process could be impaired by spider mite feeding. The down-regulation of *SAUR* gene is in agreement with the previous studies on *A. thaliana* [52] and soybean [53].

Pathogen Elicitor Perception

In our study, only one disease resistance protein from TIR-NBS-LRRs class (Phvul.005G093400.1) had higher transcript abundance in resistance cultivar, while one TIR-NBS-LRR (Phvul.010G029800.1), two NB-ARC domains-containing (Phvul.002G130666.1, Phvul.010G064700.1), and one CC-NBS-LRR (Phvul.003G247601.1) showed a high level of expression in susceptible cultivar before infestation. During the first stage of mite infestation, five TIR-NBS-LRRs (Phvul.002G323100.1, Phvul.002G323400.2, Phvul.004G046400.1, Phvul.011G140300.1 and Phvul.010G132433.1), three NB-ARC (Phvul.003G002926.1, Phvul.004G013300.1 and Phvul.008G071300.3) and one leucine-rich repeat (LRR) protein kinase (Phvul.007G087550.1) were highly expressed in resistance cultivar, whereas one TIR-NBS-LRR (Phvul.004G058700.1) and three NB-ARC (Phvul.008G031200.10, Phvul.010G064700.1, Phvul.011G195400.1) were found to be up-regulated in susceptible common bean cultivar. But these genes could not fully exert their expression with the extension of infestation time except one NB-ARC (Phvul.010G064700.1) in susceptible cultivar. At the first glance, it seems that the susceptible cultivar has a higher number of up-regulated disease resistance genes than the resistant one before mite infestation. But our results indicated that the response of the resistant plants was more robust than that of the susceptible cultivar upon pathogen attack. This can be elucidated by the role of miRNAs in down-regulating defense-related genes expression in susceptible cultivar [54, 55]. The non-specific defense responses to deter the pathogen can also explain loss of defense-related genes expression at fifth day.

In addition, one gene encoding the Cysteine-rich RLK (CRK10) was highly expressed upon spider feeding. This highly up-regulated *CRK* gene seems to indicate its potential role in resistance against spider mite. We also observed receptor-like proteins (RLPs) and receptor-like kinase (RLK) genes that have a direct effect on the pathogen in the both cultivars [56].

Antioxidant and detoxification processes

Reactive oxygen species (ROS) are involved in various processes along the plant life, but are best known as a key component of the signaling events involved in abiotic and biotic stress responses, so that are rapidly induced and accumulated after pathogen attack [57]. An important response to control ROS is the induction of scavenging genes. In this respect, heat shock proteins (HSPs) play an important role in supporting ROS scavenging activity and stress tolerance [58]. In our study, HSP 70 was found to be up-regulated during spider mite feeding only in resistant

cultivar, which highlight the function of HSPs in plant defense against pathogenic infection and reduce accumulation of ROS. This notion can be supported by the *P. vulgaris-Colletotrichum* interaction study [59], that HSPs are highly expressed against *Colletotrichum lindemuthianum* infection. Among detoxification genes, UDP-glycosyltransferase significantly up-regulated in resistant cultivar at all-time points, suggesting this gene may play an important role in the common bean resistance to spider mite feeding. The previous studies conducted on nematode attack in wheat (Qiao et al, 2018) and *Fusarium* in *Brachypodium distachyon* (Schweiger et al, 2013) reinforces our argument about UDP-glycosyltransferase function.

Cell wall

Xyloglucan endotransglucosylase/hydrolases (XTHs) are a family of enzymes that facilitate cell wall expansion [60] and also have the functions, probably associated with resistance mechanism [61]. In current study, we observed two XTHs (XTH22: Phvul.003G147700 and XTH9 :Phvul.005G111300) in resistant cultivar significantly expressed at day 1 post infestation. Another important candidate gene for spider mite resistance may be the malectin-like receptor kinase FERONIA (FER), which was up-regulated and showed increase abundance during mite infestation, although there is no significant information on the role of this gene in response to feeding.

Other genes

There are substantial reports regarding expression of pathogenesis-related (PR) genes under numerous stresses in common bean [59, 62]. Our transcriptome study successfully identified one PR-5 like receptor kinase (Phvul.004G155500) as up-regulated gene in resistant cultivar at day 5. 2-oxoglutarate (2OG) and Fe (II)-dependent oxygenase is another up-regulated gene in resistance cultivar under infestation which make it a potential candidate gene for resistance against spider mite and probably suitable for breeding programs. This gene has been previously described as responsive to pathogens [26].

Validation of DEGs by using qRT-PCR

In order to verify gene expression results of transcriptome data analysis, six DEGs having annotations were selected for qRT-PCR analysis. They include the genes encoding pathogenesis-related proteins PR5, heat shock protein, leucine rich repeat, MYB113, XTH and a WRKY50

(Fig 5). Quantitative RT-PCR analysis was conducted on 12 RNA samples that were used in the preparation of sequencing libraries. Relative expression profiles of DEGs in the both resistant and susceptible evaluated using qRT-PCR were in complete agreement with the RNA-seq data. This is in line with other studies, which showed almost the same level of fold changes between RNA-seq data and qPCR [63, 64].

Fig 5. qRT-PCR results of genes selected from the RNA-seq analysis of common bean–spider mite interaction. Expression levels of tested genes were normalized based on of *Actin* gene and then compared to relative expression values determined by RNA-seq. Relative expression values of samples were determined by using the average expression value of all replicates of a particular group. Standard deviation among replicates is represented by error bars. Res and Sus represent resistant and susceptible cultivars. TP0, TP1 and TP5 represent first, second and third time points. RT ans RS in parentheses represent qRT-PCR and RNA-seq.

Conclusion

To our knowledge, this investigation is the first study to identify molecular mechanisms involved in the common bean resistance to spider mite feeding by using RNA sequencing technology. In summary, DEGs were identified for control samples ~~and~~ 1 and [illegible] days after infestation of spider mite in resistant and susceptible cultivars of common bean. Importantly, we identified secondary metabolism, multiple disease resistance proteins, TFs and genes involved in cell wall expansion and antioxidant processes that were modulated by spider mite attack. Overall, this study extended our understanding of the defense molecular mechanisms of two common bean cultivars with different genetic backgrounds during spider mite infestation. We came to the conclusion that these data provide important and valuable information for future research in common bean.

Availability of supporting data

All RNA-Seq data were deposited to the NCBI sequence read archive (SRA) under the project PRJNA482175.

Declarations

Ethics approval and consent to participate

Not applicable.

Consent to Publication

Not applicable.

Competing interest

The authors have no competing interests to declare.

Author contribution statement

AHH, RMA, HA and MS participated in the experimental design. MS collected the samples. MS, MTM and RM performed RNA extraction. AS carried out bioinformatics analyses and wrote the manuscript. All authors reviewed and approved the final manuscript for publication.

References

1. McConnell M, Mamidi S, Lee R, Chikara S, Rossi M, Papa R, et al. Syntenic relationships among legumes revealed using a gene-based genetic linkage map of common bean (Phaseolus vulgaris L.). TAG Theoretical and applied genetics Theoretische und angewandte Genetik. 2010;121:1103-16. doi: 10.1007/s00122-010-1375-9.
2. Castro-Guerrero NA, Isidra-Arellano MC, Mendoza-Cozatl DG, Valdés-López O. Common Bean: A Legume Model on the Rise for Unraveling Responses and Adaptations to Iron, Zinc, and Phosphate Deficiencies. Front Plant Sci. 2016;7:600-. doi: 10.3389/fpls.2016.00600. PubMed PMID: 27200068.
3. Broughton WJ, Hernández G, Blair M, Beebe S, Gepts P, Vanderleyden J. Beans (Phaseolus spp.) – model food legumes. Plant and Soil. 2003;252(1):55-128. doi: 10.1023/A:1024146710611.
4. Tiwari M, Singh NK, Rathore M, Kumar N. RAPD markers in the analysis of genetic diversity among common bean germplasm from Central Himalaya. Genetic Resources and Crop Evolution. 2005;52(3):315-24. doi: 10.1007/s10722-005-5123-y.
5. Ghasemi Pirbalouti A, Golparvar G, Ali Rostampoor S. Evaluation of Seed Yield and Yield Components of Common Bean Iranian Cultivars for Inoculation with Four Strains of Rhizobium legominosarum biovar phaseoli. Journal of Agronomy. 2006;5. doi: 10.3923/ja.2006.382.386.
6. Katungi E, Sperling L, Karanja D, Farrow A, Beebe S. Relative importance of common bean attributes and variety demand in the drought area of Kenya. J Dev Agric Econ. 2011;3.
7. Capinera JL. Greenhouse Whitefly, Trialeurodes vaporariorum (Westwood) (Hemiptera: Aleyrodidae). In: Capinera JL, editor. Encyclopedia of Entomology. Dordrecht: Springer Netherlands; 2008. p. 1723-6.
8. Bensoussan N, Santamaria ME, Zhurov V, Diaz I, Grbić M, Grbić V. Plant-Herbivore Interaction: Dissection of the Cellular Pattern of Tetranychus urticae Feeding on the Host Plant. Front Plant Sci. 2016;7:1105-. doi: 10.3389/fpls.2016.01105. PubMed PMID: 27512397.
9. Kiełkiewicz M, Barczak-Brzyżek A, Karpińska B, Filipecki M. Unravelling the Complexity of Plant Defense Induced by a Simultaneous and Sequential Mite and Aphid Infestation. Int J Mol Sci. 2019;20(4):806. doi: 10.3390/ijms20040806. PubMed PMID: 30781828.
10. Sharma HC, Sharma K, Seetharama N, Ortiz R. Prospects for using transgenic resistance to insects in crop improvement. Electronic Journal of Biotechnology. 2000;3:21-2. doi: 10.4067/S0717-34582000000200001.
11. Smith C. Plant Resistance to Arthropods: Molecular and Conventional Approaches2005. 1-423 p.
12. Shoorooei M, Hoseinzadeh AH, Maali-Amiri R, Allahyari H, Torkzadeh-Mahani M. Antixenosis and antibiosis response of common bean (Phaseolus vulgaris) to two-spotted spider mite (Tetranychus urticae). Experimental and Applied Acarology. 2018;74(4):365-81. doi: 10.1007/s10493-018-0240-4.
13. Meier U. Growth stages of mono- and dicotyledonous plants: Berlin : Blackwell Wissenschafts-Verlag; 1997.
14. Bolger AM, Lohse M, Usadel B. Trimmomatic: a flexible trimmer for Illumina sequence data. Bioinformatics (Oxford, England). 2014;30(15):2114-20. Epub 2014/04/01. doi: 10.1093/bioinformatics/btu170. PubMed PMID: 24695404.
15. Schmutz J, McClean PE, Mamidi S, Wu GA, Cannon SB, Grimwood J, et al. A reference genome for common bean and genome-wide analysis of dual domestications. Nature Genetics. 2014;46:707. doi: 10.1038/ng.3008 https://www.nature.com/articles/ng.3008#supplementary-information.

16. Grbić M, Van Leeuwen T, Clark RM, Rombauts S, Rouzé P, Grbić V, et al. The genome of Tetranychus urticae reveals herbivorous pest adaptations. Nature. 2011;479:487. doi: 10.1038/nature10640 https://www.nature.com/articles/nature10640#supplementary-information.
17. Dobin A, Davis CA, Schlesinger F, Drenkow J, Zaleski C, Jha S, et al. STAR: ultrafast universal RNA-seq aligner. Bioinformatics. 2012;29(1):15-21. doi: 10.1093/bioinformatics/bts635 %J Bioinformatics.
18. Trapnell C, Roberts A, Goff L, Pertea G, Kim D, Kelley DR, et al. Differential gene and transcript expression analysis of RNA-seq experiments with TopHat and Cufflinks. Nat Protoc. 2012;7(3):562-78. doi: 10.1038/nprot.2012.016. PubMed PMID: 22383036.
19. Ghosh S, Chan C-KK. Analysis of RNA-Seq Data Using TopHat and Cufflinks. In: Edwards D, editor. Plant Bioinformatics: Methods and Protocols. New York, NY: Springer New York; 2016. p. 339-61.
20. Du Z, Zhou X, Ling Y, Zhang Z, Su Z. agriGO: a GO analysis toolkit for the agricultural community. Nucleic Acids Res. 2010;38(Web Server issue):W64-W70. Epub 2010/04/30. doi: 10.1093/nar/gkq310. PubMed PMID: 20435677.
21. Untergasser A, Cutcutache I, Koressaar T, Ye J, C Faircloth B, Remm M, et al. Primer3—New Capabilities and Interfaces. Nucleic Acids Res. 2012;40:e115. doi: 10.1093/nar/gks596.
22. Pfaffl MW, Horgan GW, Dempfle L. Relative expression software tool (REST) for group-wise comparison and statistical analysis of relative expression results in real-time PCR. Nucleic Acids Res. 2002;30(9):e36-e. doi: 10.1093/nar/30.9.e36. PubMed PMID: 11972351.
23. Ferrer JL, Austin MB, Stewart C, Noel JP. Structure and function of enzymes involved in the biosynthesis of phenylpropanoids. Plant Physiology and Biochemistry. 2008;46(3):356-70. doi: https://doi.org/10.1016/j.plaphy.2007.12.009.
24. Lea US, Slimestad R, Smedvig P, Lillo C. Nitrogen deficiency enhances expression of specific MYB and bHLH transcription factors and accumulation of end products in the flavonoid pathway. Planta. 2007;225(5):1245-53. doi: 10.1007/s00425-006-0414-x.
25. Kerio LC. The functional significance of anthocyanins in leaves: a review. Tea. 2011;32(1):46-52.
26. Galindo-González L, Deyholos MK. RNA-seq Transcriptome Response of Flax (Linum usitatissimum L.) to the Pathogenic Fungus Fusarium oxysporum f. sp. lini. Front Plant Sci. 2016;7:1766-. doi: 10.3389/fpls.2016.01766. PubMed PMID: 27933082.
27. Gao M, Wang Q, Wan R, Fei Z, Wang X. Identification of genes differentially expressed in grapevine associated with resistance to Elsinoe ampelina through suppressive subtraction hybridization. Plant Physiology and Biochemistry. 2012;58:253-68. doi: https://doi.org/10.1016/j.plaphy.2012.07.009.
28. Wang L, Wang Y, Cao H, Hao X, Zeng J, Yang Y, et al. Transcriptome Analysis of an Anthracnose-Resistant Tea Plant Cultivar Reveals Genes Associated with Resistance to Colletotrichum camelliae. PLoS One. 2016;11(2):e0148535-e. doi: 10.1371/journal.pone.0148535. PubMed PMID: 26849553.
29. Petroni K, Tonelli C. Recent advances on the regulation of anthocyanin synthesis in reproductive organs. Plant Science. 2011;181(3):219-29. doi: https://doi.org/10.1016/j.plantsci.2011.05.009.
30. Li Z, Wang J, Zhang X, Xu L. Comparative transcriptome analysis of Anthurium "Albama" and its anthocyanin-loss mutant. PLoS One. 2015;10(3):e0119027-e. doi: 10.1371/journal.pone.0119027. PubMed PMID: 25781998.
31. C Lam K, Ibrahim R, Behdad B, Dayanandan S. Structure, function, and evolution of plant O -methyltransferases. Genome / National Research Council Canada = Génome / Conseil national de recherches Canada. 2007;50:1001-13. doi: 10.1139/g07-077.

32. Grubor VD, Heckel DG. Evaluation of the role of CYP6B cytochrome P450s in pyrethroid resistant Australian Helicoverpa armigera. Insect Molecular Biology. 2007;16(1):15-23. doi: 10.1111/j.1365-2583.2006.00697.x.
33. Cifuentes D, Chynoweth R, Guillén J, De la Rua P, Bielza P. Novel Cytochrome P450 Genes, CYP6EB1 and CYP6EC1, are Over-Expressed in Acrinathrin-Resistant Frankliniella occidentalis (Thysanoptera: Thripidae). Journal of Economic Entomology. 2012;105. doi: 10.1603/EC11335.
34. Becker M, Zhang X, Walker P, Wan J, Millar J, Khan D, et al. Transcriptome analysis of the Brassica napus - Leptosphaeria maculans pathosystem identifies receptor, signalling and structural genes underlying plant resistance. The Plant journal : for cell and molecular biology. 2017;90. doi: 10.1111/tpj.13514.
35. Zhu Q-H, Stephen S, Kazan K, Jin G, Fan L, Taylor J, et al. Characterization of the defense transcriptome responsive to Fusarium oxysporum-infection in Arabidopsis using RNA-seq. Gene. 2013;512(2):259-66. doi: https://doi.org/10.1016/j.gene.2012.10.036.
36. Saslowsky D, D. Dana C, Winkel B. An allelic series for the chalcone synthase locus in Arabidopsis. Gene. 2000;255:127-38. doi: 10.1016/S0378-1119(00)00304-8.
37. Nesi N, Debeaujon I, Jond C, Pelletier G, Caboche M, Lepiniec L. The TT8 gene encodes a basic helix-loop-helix domain protein required for expression of DFR and BAN genes in Arabidopsis siliques. Plant Cell. 2000;12(10):1863-78. doi: 10.1105/tpc.12.10.1863. PubMed PMID: 11041882.
38. Xie Y, Tan H, Ma Z, Huang J. DELLA Proteins Promote Anthocyanin Biosynthesis via Sequestering MYBL2 and JAZ Suppressors of the MYB/bHLH/WD40 Complex in Arabidopsis thaliana. Molecular Plant. 2016;9(5):711-21. doi: https://doi.org/10.1016/j.molp.2016.01.014.
39. Mukhtar MS, Carvunis A-R, Dreze M, Epple P, Steinbrenner J, Moore J, et al. Independently Evolved Virulence Effectors Converge onto Hubs in a Plant Immune System Network. 2011;333(6042):596-601. doi: 10.1126/science.1203659 %J Science.
40. Fu ZQ, Dong X. Systemic Acquired Resistance: Turning Local Infection into Global Defense. 2013;64(1):839-63. doi: 10.1146/annurev-arplant-042811-105606. PubMed PMID: 23373699.
41. Weßling R, Epple P, Altmann S, He Y, Yang L, Henz Stefan R, et al. Convergent Targeting of a Common Host Protein-Network by Pathogen Effectors from Three Kingdoms of Life. Cell Host & Microbe. 2014;16(3):364-75. doi: https://doi.org/10.1016/j.chom.2014.08.004.
42. Kim SH, Hong JK, Lee SC, Sohn KH, Jung HW, Hwang BK. CAZFP1, Cys2/His2-type zinc-finger transcription factor gene functions as a pathogen-induced early-defense gene in Capsicum annuum. Plant Molecular Biology. 2004;55(6):883-904. doi: 10.1007/s11103-005-2151-0.
43. Zhu Y, Shao J, Zhou Z, Davis RE. Comparative Transcriptome Analysis Reveals a Preformed Defense System in Apple Root of a Resistant Genotype of G.935 in the Absence of Pathogen. Int J Plant Genomics. 2017;2017:8950746-. Epub 2017/03/30. doi: 10.1155/2017/8950746. PubMed PMID: 28465679.
44. Rushton PJ, Somssich IE, Ringler P, Shen QJ. WRKY transcription factors. Trends in Plant Science. 2010;15(5):247-58. doi: https://doi.org/10.1016/j.tplants.2010.02.006.
45. De Geyter N, Gholami A, Goormachtig S, Goossens A. Transcriptional machineries in jasmonate-elicited plant secondary metabolism. Trends in Plant Science. 2012;17(6):349-59. doi: https://doi.org/10.1016/j.tplants.2012.03.001.
46. Berrocal-Lobo M, Molina A. Ethylene Response Factor 1 Mediates Arabidopsis Resistance to the Soilborne Fungus Fusarium oxysporum. 2004;17(7):763-70. doi: 10.1094/mpmi.2004.17.7.763. PubMed PMID: 15242170.
47. Guerreiro A, Figueiredo J, Sousa Silva M, Figueiredo A. Linking Jasmonic Acid to Grapevine Resistance against the Biotrophic Oomycete Plasmopara viticola. Front Plant Sci. 2016;7:565-. doi: 10.3389/fpls.2016.00565. PubMed PMID: 27200038.

48. Wang W-W, Zheng C, Hao W-J, Ma C-L, Ma J-Q, Ni D-J, et al. Transcriptome and metabolome analysis reveal candidate genes and biochemicals involved in tea geometrid defense in Camellia sinensis. PLoS One. 2018;13(8):e0201670-e. doi: 10.1371/journal.pone.0201670. PubMed PMID: 30067831.
49. Chen T, Lv Y, Zhao T, Li N, Yang Y, Yu W, et al. Comparative transcriptome profiling of a resistant vs. susceptible tomato (Solanum lycopersicum) cultivar in response to infection by tomato yellow leaf curl virus. PLoS One. 2013;8(11):e80816-e. doi: 10.1371/journal.pone.0080816. PubMed PMID: 24260487.
50. Spartz AK, Lee SH, Wenger JP, Gonzalez N, Itoh H, Inzé D, et al. The SAUR19 subfamily of SMALL AUXIN UP RNA genes promote cell expansion. The Plant Journal. 2012;70(6):978-90. doi: 10.1111/j.1365-313X.2012.04946.x.
51. Markakis MN, Boron AK, Van Loock B, Saini K, Cirera S, Verbelen J-P, et al. Characterization of a small auxin-up RNA (SAUR)-like gene involved in Arabidopsis thaliana development. PLoS One. 2013;8(11):e82596-e. doi: 10.1371/journal.pone.0082596. PubMed PMID: 24312429.
52. Chen YC, Wong CL, Muzzi F, Vlaardingerbroek I, Kidd BN, Schenk PM. Root defense analysis against Fusarium oxysporum reveals new regulators to confer resistance. Scientific Reports. 2014;4:5584. doi: 10.1038/srep05584
https://www.nature.com/articles/srep05584#supplementary-information.
53. Ban Y-W, Samir Roy N, Yang H, Choi H-K, Kim J-H, Basnet P, et al. Comparative transcriptome analysis reveals higher expression of stress and defense responsive genes in dwarf soybeans obtained from the crossing of G. max and G. soja2018.
54. Navarro L, Dunoyer P, Jay F, Arnold B, Dharmasiri N, Estelle M, et al. A Plant miRNA Contributes to Antibacterial Resistance by Repressing Auxin Signaling. 2006;312(5772):436-9. doi: 10.1126/science.1126088 %J Science.
55. Li Y, Zhang Y, Shi D, Liu X, Qin J, Ge Q, et al. Spatial-temporal analysis of zinc homeostasis reveals the response mechanisms to acute zinc deficiency in Sorghum bicolor. New Phytologist. 2013;200(4):1102-15. doi: 10.1111/nph.12434.
56. Gouveia BC, Calil IP, Machado JPB, Santos AA, Fontes EPB. Immune Receptors and Co-receptors in Antiviral Innate Immunity in Plants. 2017;7(2139). doi: 10.3389/fmicb.2016.02139.
57. Mhamdi A, Van Breusegem F. Reactive oxygen species in plant development. 2018;145(15):dev164376. doi: 10.1242/dev.164376 %J Development.
58. Driedonks N, Xu J, Peters JL, Park S, Rieu I. Multi-Level Interactions Between Heat Shock Factors, Heat Shock Proteins, and the Redox System Regulate Acclimation to Heat. Front Plant Sci. 2015;6:999-. doi: 10.3389/fpls.2015.00999. PubMed PMID: 26635827.
59. Padder BA, Kamfwa K, Awale HE, Kelly JD. Transcriptome Profiling of the Phaseolus vulgaris - Colletotrichum lindemuthianum Pathosystem. PLoS One. 2016;11(11):e0165823. doi: 10.1371/journal.pone.0165823.
60. Sasidharan R, Voesenek LACJ, Pierik R. Cell Wall Modifying Proteins Mediate Plant Acclimatization to Biotic and Abiotic Stresses. Critical Reviews in Plant Sciences. 2011;30(6):548-62. doi: 10.1080/07352689.2011.615706.
61. Jiang Y, Dong J, Chen R, Gao X, Xu Z. Isolation of a novel PP2C gene from rice and its response to abiotic stresses. African Journal of Biotechnology. 2011;10:7143-54.
62. Jain S, Chittem K, Brueggeman R, Osorno JM, Richards J, Nelson BD, Jr. Comparative Transcriptome Analysis of Resistant and Susceptible Common Bean Genotypes in Response to Soybean Cyst Nematode Infection. PLoS One. 2016;11(7):e0159338. doi: 10.1371/journal.pone.0159338.
63. Liu J, Xu Y, Zhang L, Li W, Cai Z, Li F, et al. De novo assembly and analysis of the transcriptome of Rumex patientia L. during cold stress. PLoS One. 2017;12(10):e0186470-e. doi: 10.1371/journal.pone.0186470. PubMed PMID: 29023590.

64. Musacchia F, Vasilev F, Borra M, Biffali E, Sanges R, Santella L, et al. De novo assembly of a transcriptome from the eggs and early embryos of Astropecten aranciacus. PLoS One. 2017;12(9):e0184090-e. doi: 10.1371/journal.pone.0184090. PubMed PMID: 28873438.

Figure 1

Click here to download Figure Figure 1.jpg

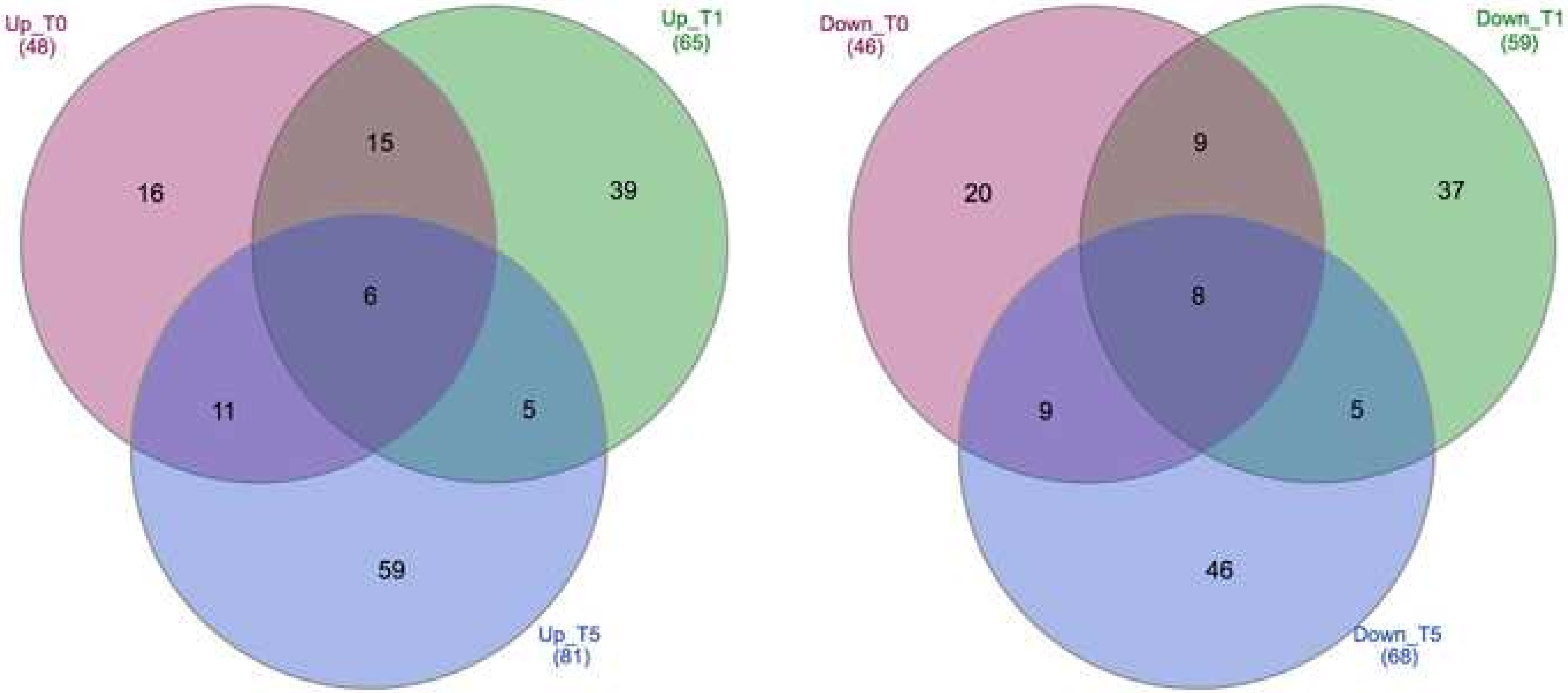

Figure 2

Click here to download Figure Figure 2.jpg

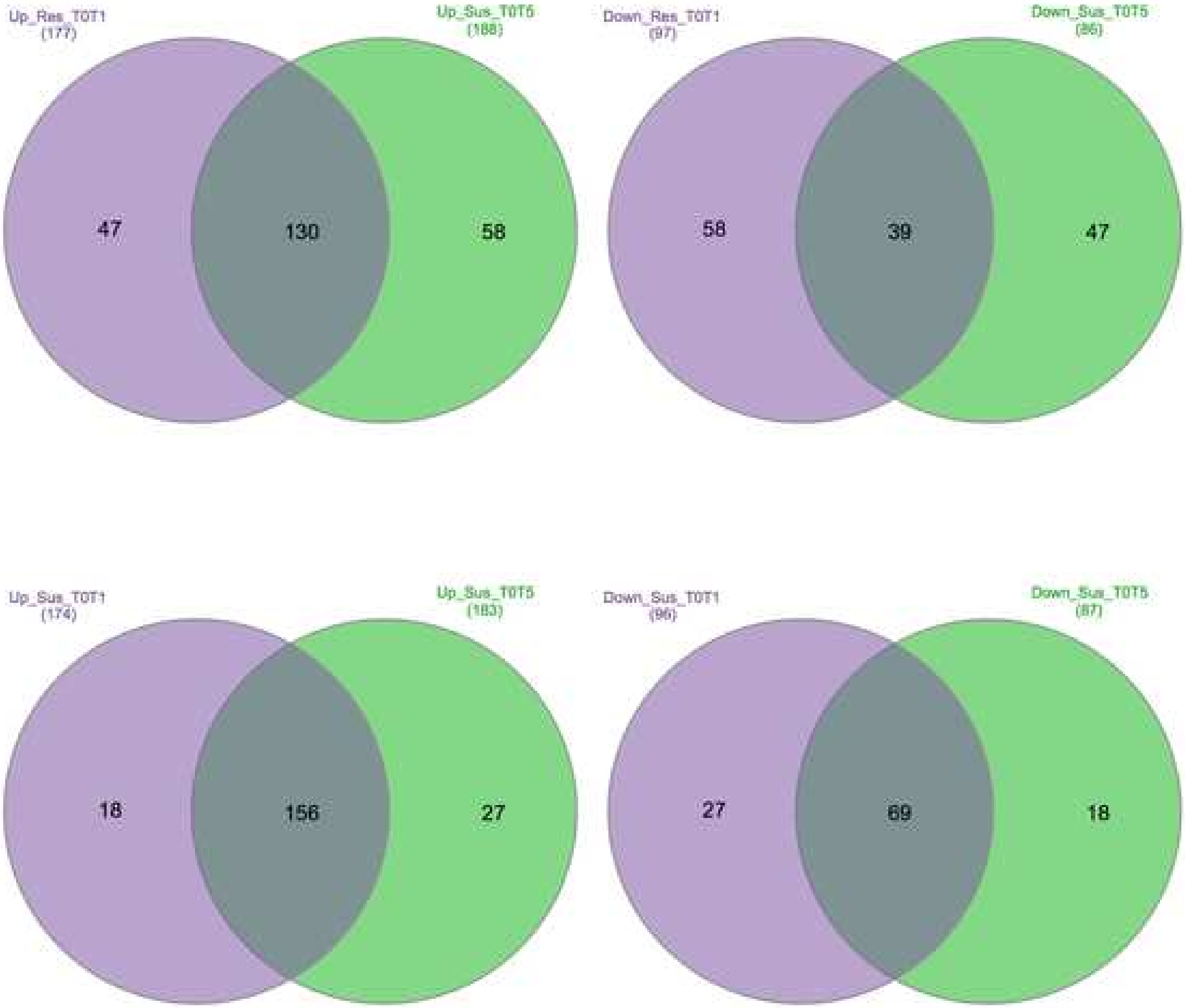

Figure 3

Click here to download Figure Figure 3.jpg

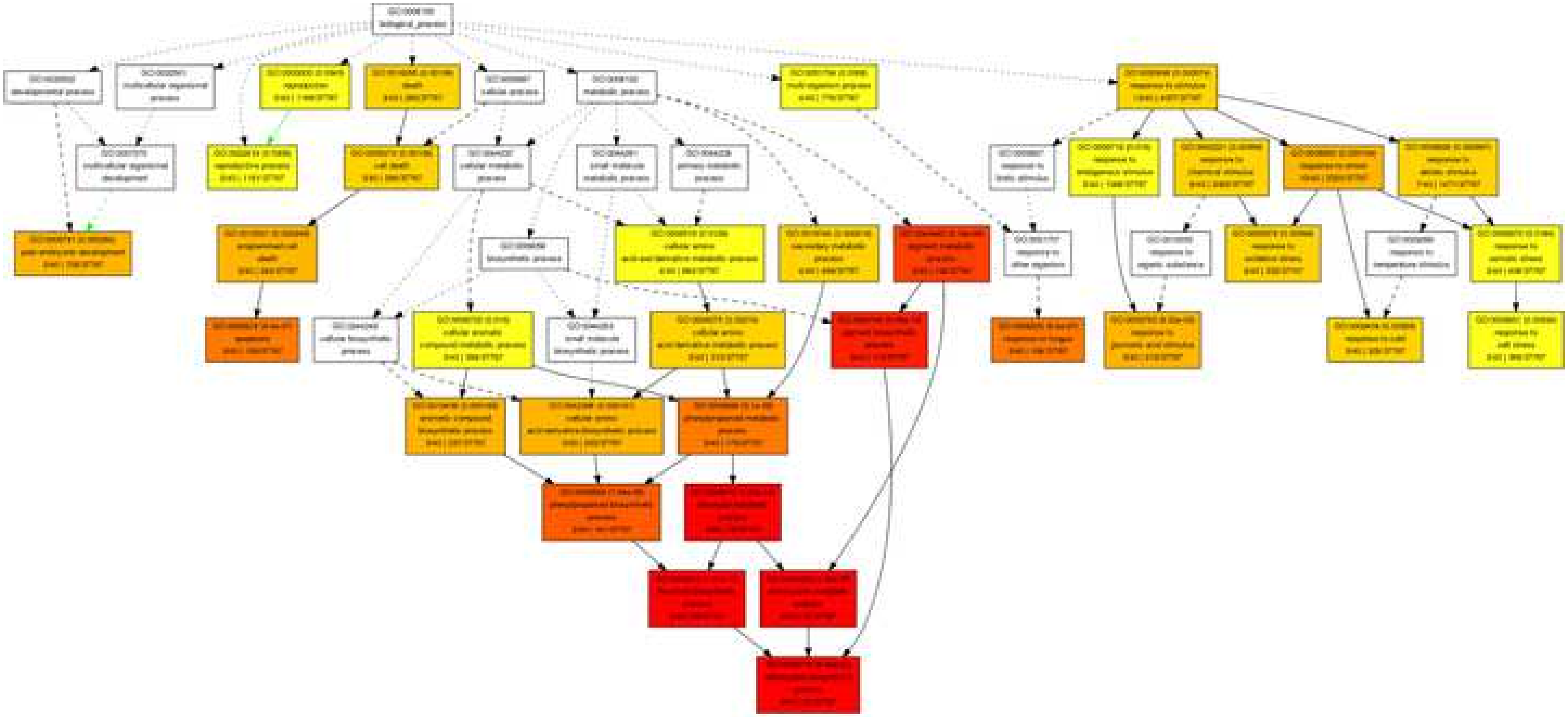

Figure 4

Click here to download Figure Figure 4.jpg

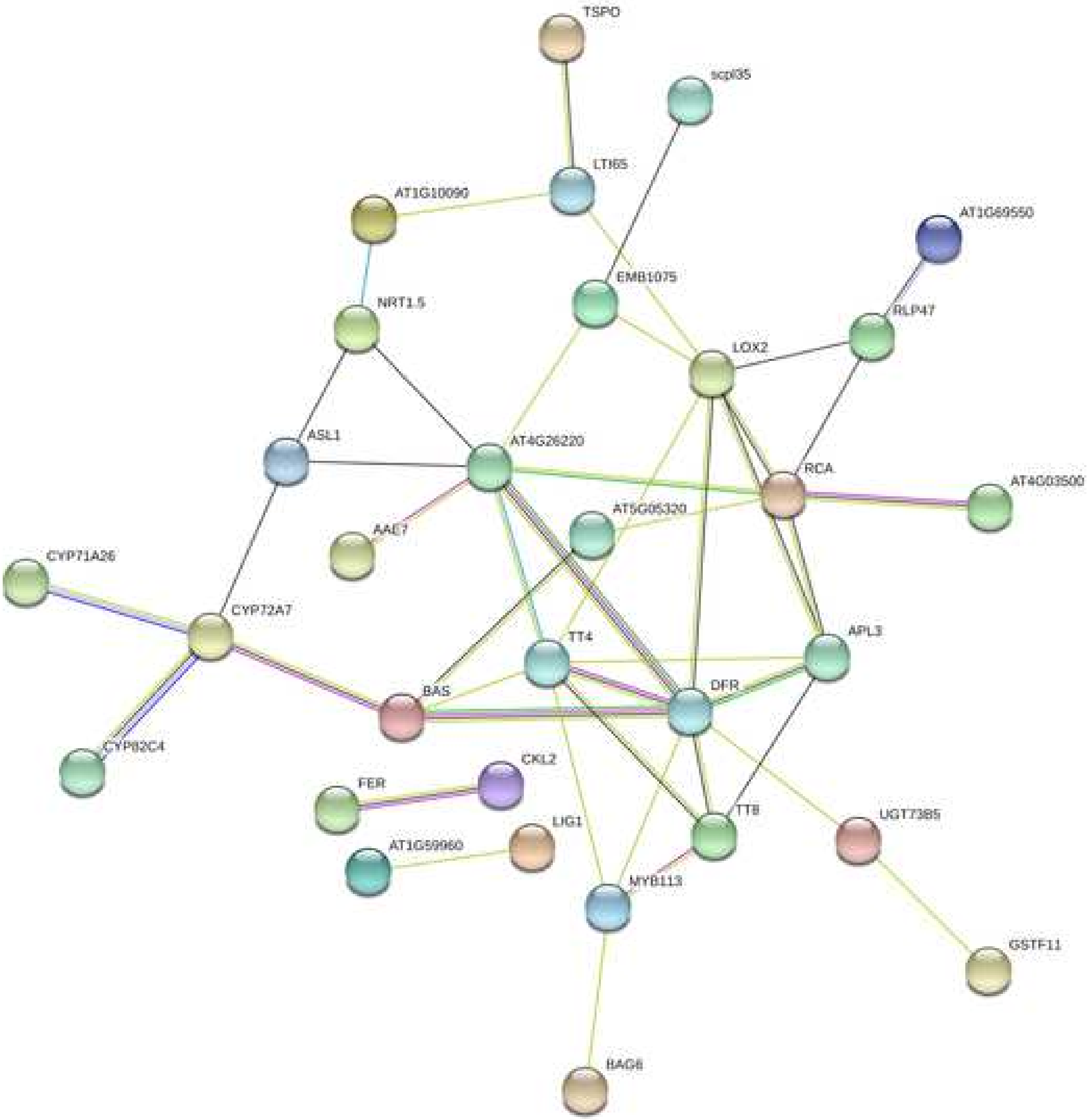

Figure 5

Click here to download Figure Figure 5.jpg

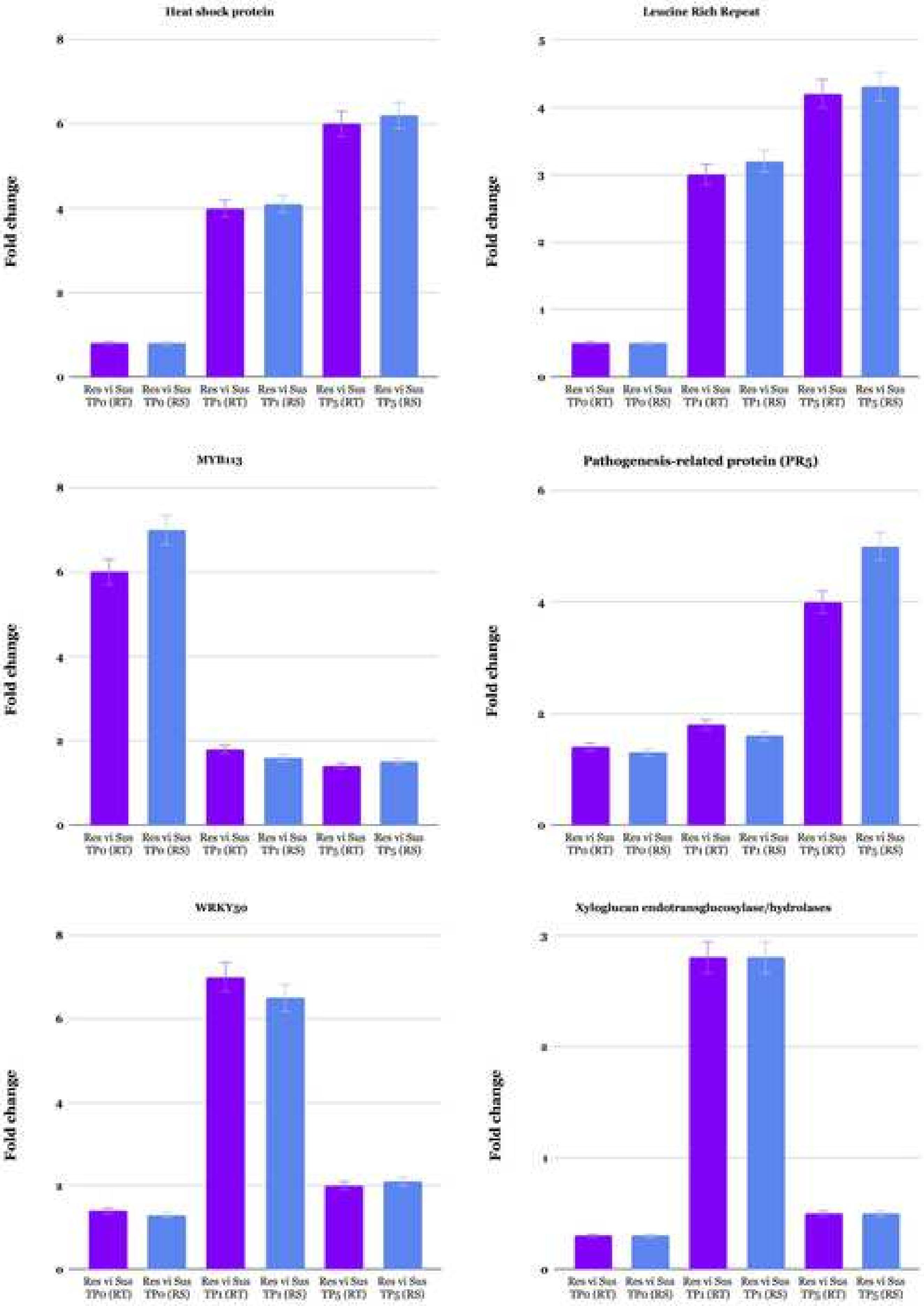

Click here to access/download
Supporting Information
Supplementary Tables.xlsx
