# Supplementary material for: Comparative transcriptome provides molecular insight into defense-associated mechanisms against spider mite in resistant and susceptible common bean cultivars
Source: PLoS One. 2020 Feb 4;15(2):e0228680. doi: 10.1371/journal.pone.0228680 (PMC6999899; doi:10.1371/journal.pone.0228680)
Supplement: Attachment — Submitted filename: Response to Reviewers.docx [file pone.0228680.s011.docx]

To: The PLOS ONE Editorial Office

Dear PLOS ONE Editor:

First of all, I would like to thank your editorial work over our manuscript. We also would like to thank the reviewers for their work and for some of the suggestions that they made. We think that the incorporation of these suggestions have increased the quality of the manuscript. We are pleased with the revision. We have addressed the concerns presented by the reviewers in the new version of the manuscript. We also have answered their questions and clarify their points in this letter. If you have any new comment or question, please do not hesitate to contact me.

Thank you in advance for your consideration,

Sincerely yours,

**Reviewer reports:**

***Reviewer #1:*** It’s known that the two-spotted spider mite Tetranychus urticae Koch is one of the economically most important pests in a wide range of outdoor and protected crops worldwide. To identify the resistant mechanism against spider mite of host plant will greatly improve the crops breeding. Authors used RNA-seq to investigate the candidate resistant related genes that’s a good idea to get the whole deferential expression genes (DEGs) between resistant and susceptible cultivars. However this manuscript lacks a systematic statement, reader hardly to read and to get the useful information. Authors may include some articles which addressed on the study on resistant mechanism of spider mite to explain the relationship of DEGs and resistant mechanism. (such as: (1) Can Plant Defence Mechanisms Provide New Approaches for the Sustainable Control of the Two-Spotted Spider Mite Tetranychus urticae?. Int J Mol Sci. 2018;19(2):614. (2) Acaricide resistance mechanisms in the two-spotted spider mite Tetranychus urticae and other important Acari: a review. Insect Biochem Mol Biol. 2010 Aug;40(8):563-72.)

For major part:

P7, Line 167: The data was not consistent to Table S3. In the manuscript the DFEs are 48 for up-regulation and 46 for down-regulation, while only 93 DFEs were included in Table S3.

>> Thank you for pointing this out. So. the missed gene was added to the list.

From P8, Line 204 to P12, Line 318: There was no related data to show the expression level of DFEs. Such as “The two most induced genes involved in flavonoid….” (Line 204), “CHS showed more than 7-fold change increase in resistant cultivar. O-methyltransferases (OMTs), involved in phenylpropanoids, flavonoids, and anthocyanin methylation, was up-regulated in resistant cultivar and showed an 8-fold increase in expression level upon spider mite feeding.” and so on. Authors need to construct some tables to indicate the expression profiles of DGEs.

>> You have raised an important point here, apologize for not making it clear. We added more information including FPKM values and log2(fold change) into Tables S1-S5 which represent DEGs between pair time points in both cultivars.

The Minor Part:

P2, Line 41: It should be “spider mite”.

>> Changed as suggested by the reviewer.

P3, Line 73: TSSM is not the abbreviation of “Tetranychus urticae Koch”, put the correct one.

>> Thank you for pointing this out. Revised!.

P4, Line 100: What does “In six-leaf stage of Meier” mean?

>> “Meier” is a surname and use as a reference. The text was changed to make it clear!

**Reference**: Meier U (1997) Growth stages of mono-and dicotyledonous plants. Blackwell Wissenschafts-Verlag.

P5, Table 5: Since there are many Gene ID related to same functional annotation, please clarify, such as “Pathogenesis-related protein, PR5” for the first item.

>> Clarified as suggested by the reviewer with adding a short explentation.

The full name should be showed at its first present, such as CYP at P8, Line 215.

>> Thanks, this point was edited.

P8, Line 216: Authors mentioned “In the susceptible genotype, CYP83B1 gene required for the synthesis of indole glucosinolates, was down regulated during infestation which is not consistent with previous reports in a number of researches”. Please explain your opinion.

>> I am grateful to the reviewer for his/her insightful comment on my paper. Thank you! This result demonstrates the role of spider mite effectors in suppressing the defence-related gene of common bean in compatible interaction (susceptible cultivar) but not in an incompatible interaction (resistant cultivar). Previous investigation indicate when a pathogen interacts with plant tissues, more intensive transcriptional changes are found in the compatible interaction and pathogens commonly develope effectors that interfere with signaling pathways to suppress resistance responses. This explentation was also added to the manuscript.

P12, Line 312: “The previous studies conducted on nematode attack in wheat (Qiao et al, 2018) and Fusarium in Brachypodium distachyon (Schweiger et al, 2013) reinforces our argument about UDP-glycosyltransferase function.” These two references were cited in a wrong format and were not included in reference list.

>> You have raised an important point here, Edited!

Reviewer #2: I think this can be an informative manuscript after adding more data to it. I added some of my recommendations as comments in the manuscript and have some more recommendations as follows: first of all, you need to perform pathway analysis and add the results to the manuscript, I recommend using KOBAS 3.0. The second thing, I already added comments on Venn diagrams, just wanted to emphasis it more here, you want to follow that and revise the manuscript accordingly. I don't see a table or graph showing the number of DEGs that you find from the comparisons that you made, you need to add it to the manuscript. I don't know if it is problem with the system that I don't see all supportive information, or something else, you do have referred to tables such as "S3-5" in the manuscript that I cannot find them. The ones that I have access to them, Table S1-S5, I don't see any column labels and title, these tables are really confusing, you need to fix it. You also want to check all gene names in the manuscript, the all need to be italicized. Your figures text is not very clear, especially Fig 5, you need to revise all of the figures and be sure that quality matches journal requirements.

>> Thank you for these suggestions. We carried out KOBAS analysis to enrich KEGG pathways and reults were provided in supplemental file as Table S6-8 for each time point comparition. Also, a new part was added into the manuscript to explain KOBAS results. Thanks for reviewer point about venn diagram. In this type of venn diagram some fields will automatically become zero which doesn’t make sense and also is too difficult/complicated to interpret. Hence we didn’t combine diagrams with 4 comparition. This would be much more complicated. All supportive information had been provided in supplementary file, however you could have access to the list of DEGs along with more details such as FPKM values and genes name. All colum lables and titles in supplementary file were edited and genes name in the manuscript were checked and italicized. We also submited high quality figures to the journal system separately. However when the system makes PDF file, figures lose quality.

Reviewer #3: The authors compared the transcriptome of resistant and susceptible common bean cultivars after infection of spider mite, aiming at shed light into the understanding of mechanisms and biological pathways of resistance. Materials and methods are not sufficiently detailed to understand the experimental design. English grammar, as it stands, needs to be improved; and some sentences are wordy and need to be shorted. Authors need to answer to the queries below, in order to resubmit to PLOS ONE for a final round and acceptance. For detailed comments, see below:

Page 4:

Lines 96-97: Information of specimen collection and rearing method of insect should be described in more detail.

>> Thank you for pointing this out. More information about this point was added into the manuscript!

Lines 97-100: Plant growth information of two cultivars should be described in more detail.

>> Plant growth condition was described in more detail.

Line 100: In six-leaf stage of Meier [13], is this phrase right? I noticed that the “Meier” is a surname.

>> Yes, “Meier” is a surname and use as a reference. The text was changed to make it clear!

**Reference**: Meier U (1997) Growth stages of mono-and dicotyledonous plants. Blackwell Wissenschafts-Verlag.

Line 100: Why only the female mites were selected? Whether these female mites were mated or not?

>> It has been demonstrated that in mite populations the number of females is higher than males and they also cause the most damages. The mean female to male ratios typically vary from 2:1 to 3:1. Also, previous studies confirm that female spider mites are able to plastically adjust sex ratio in the population. For these reasones, in our study females were selected from a mass that had the opportunity for mating. A short reason was added in text!

Line 100: The common bean cultivars growing to “six-leaf stage”, the common bean cultivars need to take how many days to grow to this stage? And I think this would be added in the text.

>> Thank you for pointing this out. It was added in the text.

Line 101: As spider mite is very small, how many individuals were used as each replicate?

>>180 adult females were used in each replicate (2 cultivar; Naz & Akhtar, 2 day; 1 & 5, and 45 mites for each leaf=2*2*45=180).

Line 101: Why the authors set three time points （0, 1and 5 days）? As I known, in some studies about defense-associated mechanisms against insect infestation, more time points (0, 12, 24, 48, and 72 h or more hours) are set after insects’ infection.

>> We agreed with the reviewer, respond to attack in some plants takes a longer infestation time (more than 3 days) but this time in some other plants in short. First day after infection was selected because, no physical damage would be visible, but we expected some metabolic changes would be occurring, and fifth day was selected because we expected, not only physical damage to visible but also metabolic changes to occur.

Reference:

1- Zhang, P. J., Zheng, S. J., van Loon, J. J., Boland, W., David, A., Mumm, R., & Dicke, M. (2009). Whiteflies interfere with indirect plant defense against spider mites in Lima bean. Proceedings of the National Academy of Sciences, 106(50), 21202-21207.

2-Prochaska, T. J., Donze-Reiner, T., Marchi-Werle, L., Palmer, N. A., Hunt, T. E., Sarath, G., & Heng-Moss, T. (2015). Transcriptional responses of tolerant and susceptible soybeans to soybean aphid (Aphis glycines Matsumura) herbivory. Arthropod-Plant Interactions, 9(4), 347-359.

Lines 100-101: What the meaning of “the same-aged”? Did the mites possess the same time span of ecdysis, forexample, 24 hours after ecdysis of these mites?

>> To obtain mites with the same age, 70 -100 gravid females from each colony were transferred on the surface of leaf discs and allowed to lay eggs for 6 h, then mites were carefully removed, hence all of the eggs are same-aged.

Lines 100-101: The sentences “45 same-aged adult female mites were placed on sixth leaf of cultivars.” Should be described in more detail. Because I was not clear how many inviduduals placed on each leaf of each cultivar per replicate.

>> 180 adult females were used in each replicate (2 cultivar; Naz & Akhtar, 2 day; 1 & 5, and 45 mites for each leaf=2*2*45=180).

Lines 105: The authors mentioned in line 98 “The experiment was conducted using a factorial experiment based on completely randomized design with three replicates”, however in line 105, they mentioned that “Total RNA was extracted from two biological replicates”. Generally, three replicates are taken for each cultivar.

>> For infestation, experiment was designed and performed with three replicates, but since RNA-Seq is still an expensive experiment, sequencing was conducted with two replicates.

Page 5:

Lines 134: the authors only six DEGs for validating candidate DEGs, I suggest that more related DEGs are need to validate.

>> We strongly agree with reviewer that a real-time PCR analysis with more genes would strengthen the paper. Nevertheless we think that this point should be more addressed in future with a higher availability of samples and more budget.

Lines 141: The 2-ΔΔCt should correct to be 2-ΔΔCT.

>> Changed as suggested by the reviewer
