# Supplementary material for: Comparative transcriptome provides molecular insight into defense-associated mechanisms against spider mite in resistant and susceptible common bean cultivars
Source: PLoS One. 2020 Feb 4;15(2):e0228680. doi: 10.1371/journal.pone.0228680 (PMC6999899; doi:10.1371/journal.pone.0228680)
Supplement: Attachment — Submitted filename: Reviewer comments.doc [file pone.0228680.s010.doc]

The authors compared the transcriptome of resistant and susceptible common bean cultivars after infection of spider mite, aiming at shed light into the understanding of mechanisms and biological pathways of resistance. Materials and methods are not sufficiently detailed to understand the experimental design. English grammar, as it stands, needs to be improved; and some sentences are wordy and need to be shorted. Authors need to answer to the queries below, in order to resubmit to PLOS ONE for a final round and acceptance. For detailed comments, see below:

**Page 4:**

1. Lines 96-97: Information of specimen collection and rearing method of insect should be described in more detail.
2. Lines 97-100: Plant growth information of two cultivars should be described in more detail.
3. Line 100: In six-leaf stage of Meier [13], is this phrase right? I noticed that the “Meier” is a surname.
4. Line 100: Why only the female mites were selected? Whether these female mites were mated or not?
5. Line 100: The common bean cultivars growing to “six-leaf stage”, the common bean cultivars need to take how many days to grow to this stage? And I think this would be added in the text.
6. Line 101: As spider mite is very small, how many individuals were used as each replicate?
7. Line 101: Why the authors set three time points （0, 1and 5 days）? As I known, in some studies about defense-associated mechanisms against insect infestation, more time points (0, 12, 24, 48, and 72 h or more hours) are set after insects’ infection.
8. Lines 100-101: What the meaning of “the same-aged”? Did the mites possess the same time span of ecdysis, forexample, 24 hours after ecdysis of these mites?
9. Lines 100-101: The sentence “45 same-aged adult female mites were placed on sixth leaf of cultivars.” should be described in more detail. Because I was not clear how many **inviduduals** placed on each leaf of each cultivar per replicate.
10. Lines 100-101: This sentence is too long, and the grammar needs to be improved.
11. Lines 105: The authors mentioned in line 98 “The experiment was conducted using a factorial experiment based on completely randomized design with **three replicates**”, however in line 105, they mentioned that “Total RNA was extracted from **two biological replicates**”. Generally, three replicates are taken for each cultivar.

**Page 5:**

1. Lines 134: the authors only six DEGs for validating candidate DEGs, I suggest that more related DEGs are need to validate.
2. Lines 141: The 2-ΔΔCt should correct to be 2^-ΔΔ^*^CT^*.
